# European Correspondence

**Published:** 1877-09

**Authors:** 


### EUROPEAN CORRESPONDENCE.

London, England, July 25th, 1877.

*Editors Atlanta Medical and Surgical Journal:*

In a series of letters I propose to give you notes and reflections on medical and surgical practice in Europe.

In framing a letter from this little world, the great difficulty is the immense amount of materials at command, so that if we were to wait to complete the round of the hundreds of hospitals, and then arrange the facts accumulated, it is quite doubtful if these communications would ever be written. Therefore, without any attempt at classification, we will note the various facts as they present themselves, and trust to the variety as a compensation for the disorder, and sometimes, perhaps, for the repetition, that will be observed.

OVARIOTOMY

is an operation surrounded with so much interest that it well deserves to be the first object of our attention, and for this we will first look at the in-department of the "Samaritan Free Hospital." As a successful operation, this one is certainly of modern origin, for some years ago, when such cases began to be received into this hospital, the ovarian operation was said to have failed; that is, the patient died, in almost every instance, at the "General Hospitals."

The Medical Superintendent of Guy's Hospital reports that, during the nine years from 1868 to 1876, inclusive, there were eighty-two cases, with thirty-nine recoveries and forty-three deaths—a mortality of 52.42 per cent. A reference to the available reports of three other large hospitals, during nine years, gives sixty-one cases, with twenty-four recoveries and thirty-seven deaths—a mortality of 60.65 per cent. In the Samaritan Hospital, for the same nine years, the mortality is only 24.37 per cent., and, in the year 1876, it has been reduced to 8.99 per cent.—fifty-five cases, with fifty recoveries and only five deaths—the lowest, it is believed, hitherto attained, or anywhere recorded.

Contrast this report with the condition of things some years ago, when, even under special arrangements the operation rarely succeeded, and the fatality, in fact, became so notorious that an inquest was threatened over every fatal case. It must be observed here, that these reports are not partial, as the Samaritan has had two hundred and eighty-one cases during the last nine years, while, for the same period of time, five large hospitals reported only two hundred and fourteen.

The success of this operasion is most desirable, because large ovarian tumors differ in one respect from many other diseases. Without the operation the patient always dies. Should she survive the operation, her restoration is as complete as if she had never been the subject of this terrific malady.

A visitor to the hospital is struck with the great precaution taken to avoid the introduction of any septic poison, each person present at an operation being required to sign a declaration to the effect that, "We, the undersigned, have not attended any post-mortem, nor any dissecting room, nor any case of infectious disease, within the last seven days." If this care is required for the success of this operation, general practitioners must of necessity resign it into the hands of specialists.

There is but one patient in each room, and the operation is performed in the same apartment in which, besides the patient's bed, there is another spare bed, and a couch for the nurse. Each case is provided with one skilled nurse, who never leaves until the end of the treatment.

There was nothing in connection with the operations but what has been described over and over again. Silk-gut was used for the sutures, to close the lower portion of the external wound, and bichloride of methylene was used for anæsthesia.

Two operations were done. One for right ovarian cystic tumor, with suppurating sack, by Dr. G. G. Bantock. In this case the sack was very adherent, and had to be carefully torn away. The pedicle was small, was simply tied and dropped into the abdomen, and a drainage tube inserted. This patient has since died,

The second case, by Mr. J. R. Thornton, was double ovarian, multiple cysts. In this the pedicles were broad and very vascular, and were laced and then dropped in. No drainage tube was used. This case is doing well.

The manner of employing the anæsthetic here, is by forcing a stream of air by means of an india-rubber hand-ball, through the fluid and into the mouth-piece. This plan, although troublesome, does away with the danger of over-administration, as the inhalation of the anæsthetic by the patient requires a positive motion on the part of the physician.

Mr. Spencer Wells, who we have seen operate twice in this hospital, is very sanguine as regards the future of ovariotomy as an operation to be as commonly performed by the general practitioner as any other capital operation—an amputation for example. Of course the opinion of Mr. Wells, as the leading ovariotomist of Europe, if not of the world, carries with it great weight, but it is not shared in by others with whom we have spoken, who regard it as an operation which must ever depend for success on careful sanitary arrangements and skilled nursing, and to this they attribute much of the success of the operation in the Samaritan Hospital, while according every credit to the skill, dexterity, and experience of the surgeons in charge.

It may be well to mention here that in case of the death of a patient *everything* in the room is removed and fumigated, and annually the hospital is closed for three weeks and thoroughly cleansed and purified.

At the Lock Hospital, for women, there are about a hundred and fifty patients, but little of novelty was presented. The mercurial treatment is employed, and inunction fully used. A patient was shown who was being treated by subcutaneous injection, and for this purpose a new preparation is under trial; it is a solution of mercurial bichloride in peptine. It is said not to precipitate, and not to cause abscesses when used hypodermically. It certainly has not produced any unpleasant effect in the patient brought forward, who had been injected daily for several days.

For eczema a new remedy, "Tong Pang Chou," is on trial. It is employed externally; but the name is not very prepossessing.

Dr. Sayre, of New York, is here, demonstrating the application of his "Plaster of Paris Cuirass," for spinal diseases. Doubtless your readers are already acquainted with the principles involved in its use, and will receive it as a great convenience to themselves, and a priceless boon to the thousands afflicted with this class of complaints.

At "King's College Hospital," after extirpating a couple of epitheliomatous tumors, Mr. Wood was kind enough to show a very interesting case of aneurism treated by electrolysis, on a new plan. After numerous trials on other cases, with not very satisfactory results, Mr. Wood conceived the idea that motion of the blood through the vessel hindered its coagulation, on the same principle that running water freezes with difficulty, and that by compression above and below the aneurism, shortly before and during the application of the galvanic current, a perfect coagulum could be produced.

The case under treatment was aneurism of the right iliac and right femoral arteries. Other means being tried without success, there remained only a choice between tying the common iliac and electrolysis, and the use of the latter was determined on. Pressure was then made above and below the tumors until pulsation ceased, needles were inserted, and a current from sixty cells passed. The effect is described as almost magical, being the complete obliteration of the vessel, so thorough, indeed, that sphacelation of the leg resulted and amputation had to be performed. The patient is doing well.

